# Case Studies on Dissocial Personality—Bad or Ill?

**DOI:** 10.3390/healthcare13010058

**Published:** 2024-12-31

**Authors:** Kasper Sipowicz, Tadeusz Pietras, Michał Sobstyl, Anna Mosiołek, Monika Różycka-Kosmalska, Jadwiga Mosiołek, Ewa Stefanik-Markowska, Michał Ring, Krystian Kamecki, Marcin Kosmalski

**Affiliations:** 1 Clinic and Polyclinic of Geriatrics, National Institute of Geriatrics, Rheumatology and Rehabilitation, 02-637 Warsaw, Poland; kasper.sipowicz@spartanska.pl (K.S.); tadeusz.pietras@umed.lodz.pl (T.P.); 2 Department of Clinical Pharmacology, Medical University of Lodz, 90-153 Lodz, Poland; 3 Neurosurgery Department, Institute of Psychiatry and Neurology in Warsaw, 02-957 Warsaw, Poland; mrsob@op.pl; 4 Department of Interdisciplinary Research in the Area of Social Inclusion, The Maria Grzegorzewska University in Warsaw, 02-353 Warsaw, Poland; manitka@tlen.pl; 5 Department of Elektrocardiology, Medical University of Lodz, 92-231 Lodz, Poland; monika.rozycka-kosmalska@umed.lodz.pl; 6 Department of Psychiatry, Medical University of Warsaw, 02-091 Warsaw, Poland; jadmosiolek@gmail.com; 7 Second Department of Psychiatry, Institute of Psychiatry and Neurology in Warsaw, 02-957 Warsaw, Poland; stefanicha@o2.pl (E.S.-M.); michalring@gmail.com (M.R.); kdjkamecki@gmail.com (K.K.)

**Keywords:** dissocial personality, social rehabilitation, case studies, genetic factors, environmental factors

## Abstract

Dissocial personality is understood as a personality that does not ideologize most social norms and is characterized by a lack of empathy. Precise criteria for diagnosing dissocial personality are included in the ICD-10 classification, which is still in force in Poland. This classification is widely available in both Polish and English. In Poland, there is a fairly wide range of assistance available for people with personality disorders in day care units and 24-h wards for the treatment of personality disorders. Unfortunately, due to some antisocial behaviors that violate the criminal law in force in Poland, people with dissocial personality are placed in prisons. The development of dissocial personality depends on both genetic factors and the demoralizing influence of the social environment. The mutual interactions of genetic and environmental factors in the pathogenesis of dissocial personality can be analyzed both using statistical methods for large groups and by analyzing a case study, which is a qualitative study and is underestimated in modern medicine. Due to the complex pathogenesis of dissocial personality, various ethical dilemmas arise, and the extent of the guilt for the committed, prohibited act depends on genetic factors and brain structure and to some extent on environmental factors. The apparent ability of people with dissocial personality to look into their own actions leaves doctors always with the question of how sick or bad the person is. In this study, we used the method of qualitative analysis of case studies of two patients treated in a 24-h personality disorder treatment unit of the Department of Neuroses, Personality Disorders and Eating Disorders of the Second Psychiatric Clinic of the Institute of Psychiatry and Neurology in Warsaw.

## 1. Introduction

Dissocial personality affects 2–3% of the society, with a 3:1 ratio of men to women [1]. It is characterized by a persistent pattern of breaking social rules and disregarding other people, accompanied by a tendency for impulsiveness and lies, irresponsibility, low empathy, and establishing temporary, random relationships [2]. Such people are believed to often come into conflict with the law. In prisons, 47% of men and 21% of women meet the criteria for dissocial personality [3]. However, not all the individuals affected by this disorder come into conflict with the law. There is a subgroup of the so-called “successful psychopaths” who can control pathological behavior in order to protect themselves from the looming legal consequences. “Successful psychopaths” can sometimes achieve great success in business, science, medicine, or politics [1].

Like in the case of most mental disorders, the causes of dissocial personality are believed to depend on both genetic and environmental factors. Psychosocial factors interact with biological factors in shaping aggressive/criminal behaviors [4,5]. Moreover, changes in personality could be potentially linked to the amount of substance abuse, such as heroin [6]. There is no confirmed evidence that predispositions or neurobiological changes are solely responsible for dissocial personality [7]. Studies on twins have shown that heritability amounts to 38–69% [8]. Environmental factors include violence and sexual abuse of a child, poor quality of parent–child relationships, poor parenting skills, nuclear family violence, modeling of a child’s behavior by their parents with psychopathic characteristics and by peers exhibiting behavioral disorders, frequent viewing of violent videos, and brain microtraumas sustained in childhood, e.g., as a result of beating [9,10]. Research shows that approximately 6% of criminals commit the majority of all crimes [11], and in 5% of families, there are more than 50% of all arrests [12]. Aggressive behavior and a tendency to violence, like other forms of human behavior, occur under specific genetic and environmental conditions and require their interaction. Aggression is usually the ultimate result of a chain of life events, during which risk factors for criminal behaviors accumulate and potentially amplify.

Having a dissocial personality is not a matter of the individual’s conscious choice, but a permanent syndrome of traits inherited or acquired as a result of educational errors or the environment [8,13,14]. Therefore, an ethical question arises whether such persons should be liable for the committed offenses. On the other hand, persons with dissocial personality who have the correct quotient of intelligence are able, from an intellectual point of view, to predict the consequences of the acts committed.

Therefore, the aim of our study was the idiographic analysis of the moral dilemma of the bad or ill based on the example of case studies.

## 2. Materials and Methods

An idiographic qualitative analysis of 2 case studies of people with dissocial personality was undertaken in this study. The patients’ life stories were analyzed for genetic and environmental risk factors for the formation of dissocial personality using the hermeneutic method. This analysis prompted reflection on the ethical aspects of punishing/treating people with dissocial personality. Dissocial personality was diagnosed using the ICD-10 research criteria. In Poland, the classification that is still in force is ICD-10. Therefore, in this study, the ICD-10 research criteria were used to diagnose the disorder. In DSM-5, dissocial personality is called antisocial personality, and the nosological criteria of which are almost identical to the research criteria according to ICD-10. This means that both of our cases meet both the nosological criteria of dissocial personality according to ICD-10 and the nosological criteria of antisocial personality according to DSM-5.

In both of our case studies, patients were treated with analytic group therapy and therapeutic community therapy in a 24-h personality disorder treatment unit of the Department of Neuroses, Personality Disorders and Eating Disorders of the Second Psychiatric Clinic of the Institute of Psychiatry and Neurology in Warsaw.

## 3. Results

### 3.1. Case Number 1

The patient K.W., aged 30, was admitted to the Department of Neuroses, Personality Disorders and Eating Disorders of the Second Psychiatric Clinic of the Institute of Psychiatry and Neurology in Warsaw for the treatment of personality disorders. The patient had been raised in an incomplete family—their alcoholic father was imprisoned for committing a murder with particular cruelty when the child was 1 year old. He was brought up by his mother and grandmother. He had never met his father’s family. He also bore his mother’s maiden name. According to the mother’s narrative, the family of the patient’s father are criminals and alcoholics. In the elementary school and junior high school, he demonstrated ADHD (attention deficit hyperactivity disorder) traits and wetted the bed at night. He remained under the care of a psychological and pedagogical counseling center. He was a child rejected by his peers, always playing alone, and often becoming a victim of peer violence. He was in elementary school and was already fascinated by the killing of animals, and he felt special pleasure in strangling small kittens. He also killed frogs and lizards, which he claimed was because of his interest in biology and his desire to see their internal organs. He also liked to tease younger children, especially his cousins—a boy and a girl. His mother and grandmother were deeply religious persons. As a result, they forced the patient to be an altar boy. He was happy to go to church because he was accepted by other acolytes, which gave him a sense of participation in a peer group. He also reported that, at the age of 13, he was forced by a priest to participate in oral and genital sex. This was the period when he and his fellow acolytes discovered pornography. Watching pornography, they masturbated each other and for this reason often went to confession. The patient’s addiction to masturbation, in which he indulged a dozen or so times a day, was accompanied by guilt and fatigue. He noticed that he was attracted to both women and men, but could not find a permanent girlfriend or partner. He masturbated, imagining his classmates, both girls and boys, in violent scenes, for example, after the intercourse, he cut off their heads. In senior high school, the symptoms of ADHD decreased, and he began to learn well. However, he liked to smoke and use marijuana, cocaine, ecstasy, and occasionally heroin. He took addictive substances irregularly, fearing the consequences. At weekends, he often got drunk and did not come home for the night. When he was in senior high school, he began using the services of prostitutes. He felt special sensations while playing scenes of violence with them. He sometimes earned money from an older sponsor who paid him for sexual services. Sometimes, wandering at night, he robbed homeless and intoxicated people. He went to a camp organized by the church, where his task was to take care of two autistic boys. In the evening, he masturbated kissing one of them, who did not speak. According to the patient, he was extremely delighted at the time. When questioned, he showed no guilt due to the sexual exploitation of a minor with intellectual disabilities and general developmental disorders. He claims that he showed him the charms of sex, and that the boy himself demanded it, and besides, because of the lack of speech development, he would not tell anyone about it anyway. After graduation, he decided to join the seminary conducted by Order X. There, after a period of good adaptation, he began to drink alcohol and take drugs, for which he was expelled from the seminary. After leaving the seminary, he enrolled in a university-profile theological course, which he completed on time, despite suspicions that he had robbed one of his colleagues. After his undergraduate studies, he began teaching catechesis at a primary school in the city where he came from. At that time, in one of the parsonages, he drank alcohol with two priests every weekend and indulged in various sexual experiments with them. After several years spent as a teacher, he decided to rejoin the seminary, this time at the level of the diocese. There, he was observed several times drinking alcohol and taking drugs. For that reason, he was referred to an outpatient psychiatric department. In that department, a full psychological and psychiatric diagnosis was performed. The Wechsler test found that the patient’s intelligence quotient was higher than the average (120) with balanced development in the verbal and nonverbal scales. In a neuropsychological examination, he demonstrated significant deficits in attention and certain executive functions. Based on personality assessment tools, including MMPI (Minnesota Multiphasic Personality Inventory), he was diagnosed with dissocial personality, with significantly marked sadism and a lack of empathy. The patient understood social situations perfectly, but could not sympathize with other people or victims of violence. Fantasizing about another person’s sufferings gave him pleasure akin to sexual pleasure. At the same time, as a religious person, he fears hell.

### 3.2. Case Number 2

The patient A.L., aged 34, was admitted to the clinic to undertake group psychotherapy. The patient was born into an incomplete family. He lived with his grandmother, mother, and two sisters, one two years older and the other three years younger. Each child had a different father. The mother never got married. She drank alcohol regularly. She brought home men, with whom she had sexual intercourses in the presence of her children. The patient suspects that his mother prostituted herself. The children were brought up by their grandmother, whom the patient remembers as a warm and caring person. The older sister physically abused her younger siblings. The moment the patient gained physical fitness he began to harass both of his sisters. He had his first sexual intercourse with his own sister at the age of 14. He says it was a single incident because he felt disgust for her. The patient was a truant who did not learn and got into fights. For this reason, he had a probation officer for 4 years. At the age of 18, he left the family home and started to live with a concubine who was 10 years older than him, with whom he had a child (son). At that time, he had no conflicts with the law and graduated from the extramural secondary technical school, obtaining the final examination certificate with very poor grades. The patient prided himself on completion of that school, as he was the only person in the family with secondary education. At the age of 25, he left his partner. The court obliged the patient to pay alimony for his son, which he did irregularly. At the age of 23, he took up courier work, and he lost that job after 4 years due to suspected theft and high absenteeism. The patient denies that he robbed a client, claiming that he was the victim of a conspiracy involving his former partner and his employer. For the alleged theft, the court sentenced him to imprisonment, and the execution of which was suspended for the probation period.

It was a pleasure for the patient to observe harmless car accidents, of which he was an indirect perpetrator himself, spilling engine oil on the roadways. Several times he deliberately pushed older people down the escalator and poured water onto the outdoor stairs in winter to cause icing and slipping of passing people.

The patient was formally registered for permanent residence with his grandmother, but he did not stay there regularly because she “was always preaching” and refused to give him money. On several occasions, he robbed his grandmother and beat his younger sister once. During a psychological examination, the patient demonstrated correct auto- and allopsychic orientation and no disturbances of the cognitive processes, and he scored 98 points as an intelligence quotient score measured by WAIS-R (PL), namely, the Wechsler Adult Intelligence Scale—the revised, Polish version. The examination using the SCID-2 questionnaire revealed dissocial personality. The patient understands the meaning of emotions, but he cannot feel any empathy for other people. He considers himself a victim of the family home, school, concubine, and the state. For this reason, he has a depressed mood and feels unhappy. He exhibits primitive defense mechanisms of personality. When asked if he felt pity for the victims of the road traffic collision that he had caused by spilling oil, he claimed that he had not killed anyone, and nothing had happened to anyone, and other people had done worse things.

A summary of the likely genetic, common, and specific environmental factors in the analyzed patients are presented in Table 1.

## 4. Discussion

Modern neuroscience and cognitive science assume that people differ in terms of cognitive performance, emotional reactivity, preferences, and behavior. This phenomenon is called neurodiversity [15]. Neurodiversity is determined by the product of genetic factors as well as by specific and common environmental factors. For example, intelligence measured using the Wechsler test depends on the genetic factor variation [16] in about 50% of cases, while some mental disorders are dependent on the genetic factor in approximately 70% of cases [17,18]. According to various studies, the inheritability of dissocial personality amounts to 38–69% [8]. Mental disorders and behavioral disorders are considered to be those syndromes of mental and behavioral characteristics of an individual that are considered dysfunctional regardless of the social context at any specific time in the cultural development of civilization. An example is the breakdown of the lifeline in the course of psychosis, or the incapability of social adaptation in subjects with a non-normative personality. These phenomena, considered by science as abnormalities, are classified in ICD-10 and DSM-5 as specific nomothetic category recognition classes. Modern psychiatry strives for social inclusion of different individuals. This is implemented by standardizing the social environment in such a way that as many people as possible, regardless of their neurodiversity, can find their place in society. This demand coincides with modern trends in special pedagogy (education) [19]. Its relative utopianism means that certain extreme varieties of neurodiversity will always be perceived by a part of the society as abnormal and threatening [20]. A mature civil society develops a system of social control over such behavior, which is usually regulated by the doctrines of the national penal codes [21]. Individuals with dissocial personality account for a significant proportion of people isolated in prisons [22]. By design and social practice, such isolation is usually a punishment, and not a corrective action, i.e., therapy. Therefore, a question arises whether persons burdened with genetic pathological behavior and exposed to domestic violence (a common environmental factor) and external social pathology should be punished. Where were the institutions of the state, social welfare, schools, and religious associations when the creation of conditions for a person burdened with genetically determined antisocial behavior to develop this psyche was to be prevented? It is assumed that people with psychosis have no insight into their own behavior [23]. Therefore, they cannot be punished for the criminal acts committed (in Poland, Art. 31, §1,2,3 of the Criminal Code dealing with the exclusion from criminal liability of persons with mental disorders). However, the question arises whether a person with dissocial personality is able to control consistently his/her conduct in such a way as not to harm others [24]. Intellectually, each of the described subjects was able to understand cause-and-effect relationships, understood what good and evil are, knew the consequences of their own behaviors, and thus the impact on their conduct. However, even if it is assumed that a particular patient has a partial insight into the effects of his/her conduct, can he/she be blamed for such mental predispositions? It seems that the assessment of the level of intellect without taking into account other factors is very one-dimensional. It is noteworthy that the legislators are divided in their opinions on people with psychoses and those with abnormal personalities. If it is assumed that prison isolation cuts off the majority of society from the negative effects of the conduct of persons with dissocial personality, then the question must be asked whether such isolation among the psychopathic personalities gathered in prison brings any results? Rehabilitation theorists themselves emphasize that prison isolation is not the best way to deal with people who repeatedly violate the social norms [25]. An alternative may be probation, which, however, does not have developed safety standards for the majority of the population [26,27]. An indirect method between isolation and probation is electronic surveillance of the person serving the sentence in the home conditions.

Research into twins demonstrates the genetic impact on the development of dissocial personality [28,29]. An indirect evidence of the genetic nature of these disorders is provided by the presence of typical anatomical changes in the brain, confirmed by functional magnetic resonance imaging [14]. The most commonly reported abnormalities affect the amygdala, prefrontal cortex, and temporal cortex [30,31,32]. These CNS structures are smaller and show reduced activity. Anatomical changes primarily include the ventral system connecting the anterior temporal lobe to the anterior and ventral frontal areas and the dorsal system connecting the middle frontal lobe with the posterior cingulate gyrus system and with the medial structures of the temporal lobe. CNS functional imaging data indicate a reduction in the activation of both systems during the challenge, suggesting the existence of specific mechanisms by which emotion is incorrectly integrated into cognitive processes [33]. Particularly, severe antisocial features were observed in people with hypoactivity of the HPA axis (hypothalamic–pituitary–adrenal stress axis) [34,35].

An equally important contribution to the formation of dissocial personality is the specific environmental factor [8]. One of the mechanisms that contributes to abnormalities in the development of the CNS is chronic stress, which causes an increase in corticosteroid levels, which can lead to apoptosis [36]. Children whose nervous system is still undergoing numerous changes seem to be particularly susceptible to the pro-apoptotic and plasticity-inhibitory properties of cortisol [37], which affects processes such as myelination, the development of neural networks, and neurogenesis [38,39]. The long-term, toxic effects of cortisol on neurons are persistent [40]. Andersen et al. reported that sexually abused children are characterized by changes in the frontal lobe and the corpus callosum, which is the largest band of white matter and integrates the work of both hemispheres [37]. Changes caused by chronic stress can be observed not only in the structure but also in the function of the reward system. People who were exposed to stress in childhood are characterized by a weakened positive response to the reward [41].

Assuming that the task of the education process is to shape the personality of the wards, the question should be asked who made the mistake, thus failing to attempt to correct the patient’s behavior sufficiently, if it was possible. Another problem is the identification of people at risk of developing dissocial personality. The broader problem is whether any prevention programs are developed at all to prevent the development of antisocial behavior in genetically predisposed persons. It is no big deal to punish but is to prevent and counteract the commitment of socially harmful offenses. In the light of these data, the question arises whether anybody can be blamed for their gene set, brain structure, or social neglect in their upbringing [42]. On the other hand, people with dissocial personality have the correct intelligence quotient to predict the effects of their own behavior, although they show numerous cognitive deficits [14]. The difficulty in interiorizing social norms as the subject’s own norms must not be a mitigating factor. In the reflection on the guilt/non-guilt of persons with dissocial personality in criminal proceedings, we come to an aporetic contradiction of doctrines, concepts, and attitudes. There is currently no conclusive research inferring that judgment, insight, and the ability to direct one’s behavior are impaired in any personality disorder. At the moment, this problem is unsolvable and really depends on the structure and doctrine of criminal law in a particular country and cultural area [43,44].

## 5. Conclusions

Currently, dissocial personality is known to develop as a result of the interaction of genetic and environmental factors [11]. Forensic psychology, social rehabilitation, and criminal law are dominated by the discourse on punishing such persons in the form of prison isolation for criminal acts committed. The careful analysis of the phenomenon suggests that the development of dissocial personality should be first of all prevented and, if it has already developed, the scientifically proven standards for the treatment of this disorder should be utilized. This ideal premise means that, in the distant future, prison isolation should become a marginal form of protecting healthy people from the effects of aggression from individuals with dissocial personality. Such a shift in paradigms is possible. An example is the phenomenon of non-heteronormative orientation, which in the 19th century was subjected to prison criminalization, followed by forced treatment, classification as a perversion, and then followed by voluntary treatment by methods of conversion therapy. Currently, this phenomenon is considered to be a variant of the norm, and any attempts to change it are considered unethical by psychiatrists and psychologists [45]. Therefore, we believe that a similar evolution will occur for people with dissocial personality, provided that effective methods of prevention and correction are developed.

## Figures and Tables

**Table 1:** A summary of the likely genetic, common, and specific environmental factors in the analyzed patients.

Factor	Patient K.W.	Patient A.L.
Probable genetic factor	A father in prison sentenced for murder; ADHD and bed wetting.	Dissocial personality traits of the mother and probably the father; Features of the incorrectly shaped personality of the older sister.
Probable common environmental factor	Inoperable educational environment of the patient’s family home; Lack of father and male pattern of behavior.	Inoperable educational environment of the patient’s family home; Prostitution of the mother; Lack of father and male pattern of behavior; Nuclear domestic violence.
Probable specific environmental factor	Rejection at school by peers; Victim of sexual exploitation at the age of 13; Viewing pornographic content during adolescence; Use of addictive substances and being a truant; Use of paid forms of sex; Prostitution.	Features of the incorrectly shaped personality of the older sister; Incest in the family; Being a truant, and non-compliance with compulsory schooling; Social contacts with peers with similar behavioral characteristics as the patient.

ADHD—attention deficit hyperactivity disorder.

## Data Availability

The data presented in this study are available on request from the corresponding author. The data are not publicly available due to ethical reasons.
